# Supplementary material for: Architecture of a mammalian glomerular domain revealed by novel volume electroporation using nanoengineered microelectrodes
Source: Nat Commun. 2018 Jan 12;9:183. doi: 10.1038/s41467-017-02560-7 (PMC5766516; doi:10.1038/s41467-017-02560-7)
Supplement: Supplementary file 1 — Supplementary Information [file 41467_2017_2560_MOESM1_ESM.pdf]

Supplementary Figures

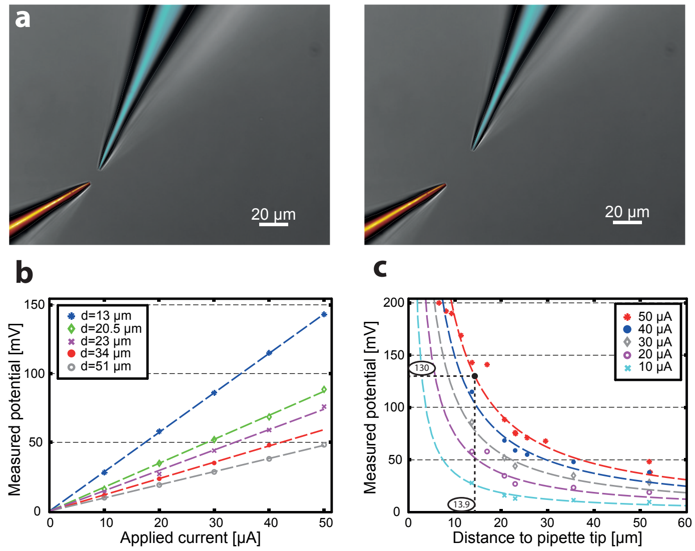

Supplementary Figure 1: Experimental validation of the FEM model

(a) Experimental setup as seen under the 20x water-immersion objective. The stimulating electrode (false coloured in yellow-orange) was kept in the same position while the recording electrode (false coloured in cyan) was moved to different positions in the same plane to measure the potential at varying distances *d*. Left image shows the position at 11 µm distance to the tip of the stimulating electrode, while the recording electrode in the right image is located at a distance of 29 µm. **(b)** Measured potential plotted against the applied current at five distances *d*. Dashed lines are linear fits through the origin (*d* = 34 µm red, R^2 = 0.9994; *d* = 13 µm blue, R^2 = 0.9999; *d* = 51 µm gray, R^2 = 0.9996; *d* = 20.5 µm green, R^2 = 0.9991; *d*= 23 µm magenta, R^2 = 0.9990). **(c)** Measured potential as a function of distance to the tip of the stimulating electrode for five different stimulus intensities. Dashed coloured lines are fits according to the potential V of a point current source I_0 where $V = I_0/(4\pi\sigma d)$, with saline conductivity σ being the only free parameter (R^2 = 0.9134, σ = 2.142 S m^{-1}). The black dashed lines indicate the critical distance (13.9 µm) where the assumed electroporation threshold of 130 mV (+70 mV for the resting membrane potential) is reached.

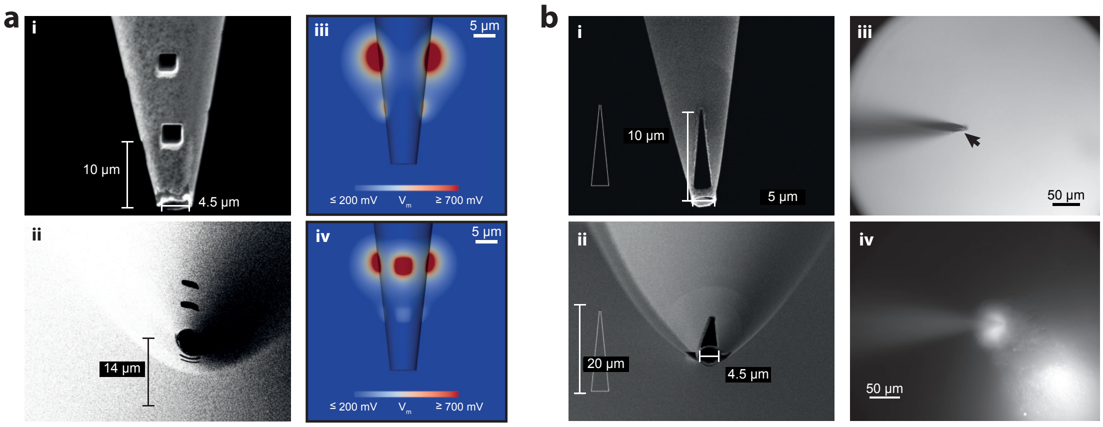

Supplementary Figure 2: Examples of other pipette designs and numerical modelling

(a) Examples of a pipette featuring a 2x2 and 2x4 design. (i) Example of an NEM after successful insertion of the two-level hole design at 10 and 20 µm from the tip with an edge length of 3 µm and 2.8 µm, respectively, as seen in high-resolution FIB imaging mode. (ii) Corresponding top view in the SEM mode. (iii) Corresponding cross-section of the 3D-FEM model illustrating total effective electroporation volume and its distribution around the pipette tip at 50 µA employing the 2x2 design. (iv) Same representation for the case of two release levels and holes from all four sides. **(b)** Example of physical damage when employing large release sites. (i) Example of an NEM after successful insertion of a triangular hole design extending from 2 µm above the tip to 20 µm proximal from the tip, as seen in high-resolution FIB imaging mode. (ii) Corresponding top view in the SEM mode. (iii) light-microscopic image showing the inserted release site prior to insertion into an acute brain slice. (iv) green fluorescent microscopic image showing the pipette tip after retraction from the *MOR-174-GFP* glomerulus in an acute brain slice removing large parts of the glomerular tissue.

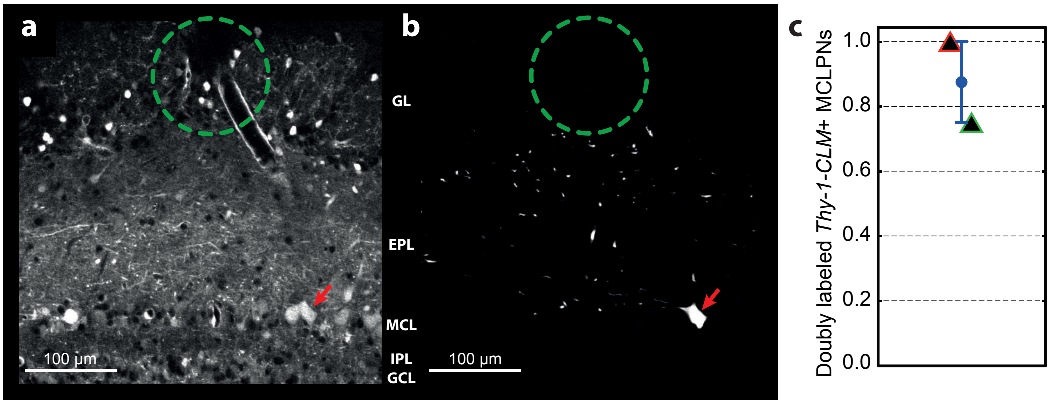

Supplementary Figure 3: *Thy-1-CLM* Electroporation

(a) *Thy-1-CLM* fluorescence of the olfactory bulb region as seen in a CLSM imaging plane next to the electroporation site. Red arrow indicates a *Thy-1-CLM* positive MCLPN which is morphologically found to be connected to the electroporated glomerulus. **(b)** Corresponding TMR fluorescence after electroporation. The marked MCLPN from (a) (red arrow) also shows positive TMR labeling while the adjacent, *Thy-1-CLM* positive MCLPN projecting to a different glomerulus remains TMR negative. **(c)** Fraction of TMR-loaded *Thy-1-CLM*-positive MCLPNs which show affiliation with the electroporated glomerulus (n=2). Blue bar and circle indicate mean value and standard deviation. Dashed green circle representing approximate location of the electroporated glomerulus in a nearby z-plane of the imaging stack. GL = glomerular layer, EPL = external plexiform layer, MCL = mitral cell layer, IPL = internal plexiform layer, GCL = granule cell layer.

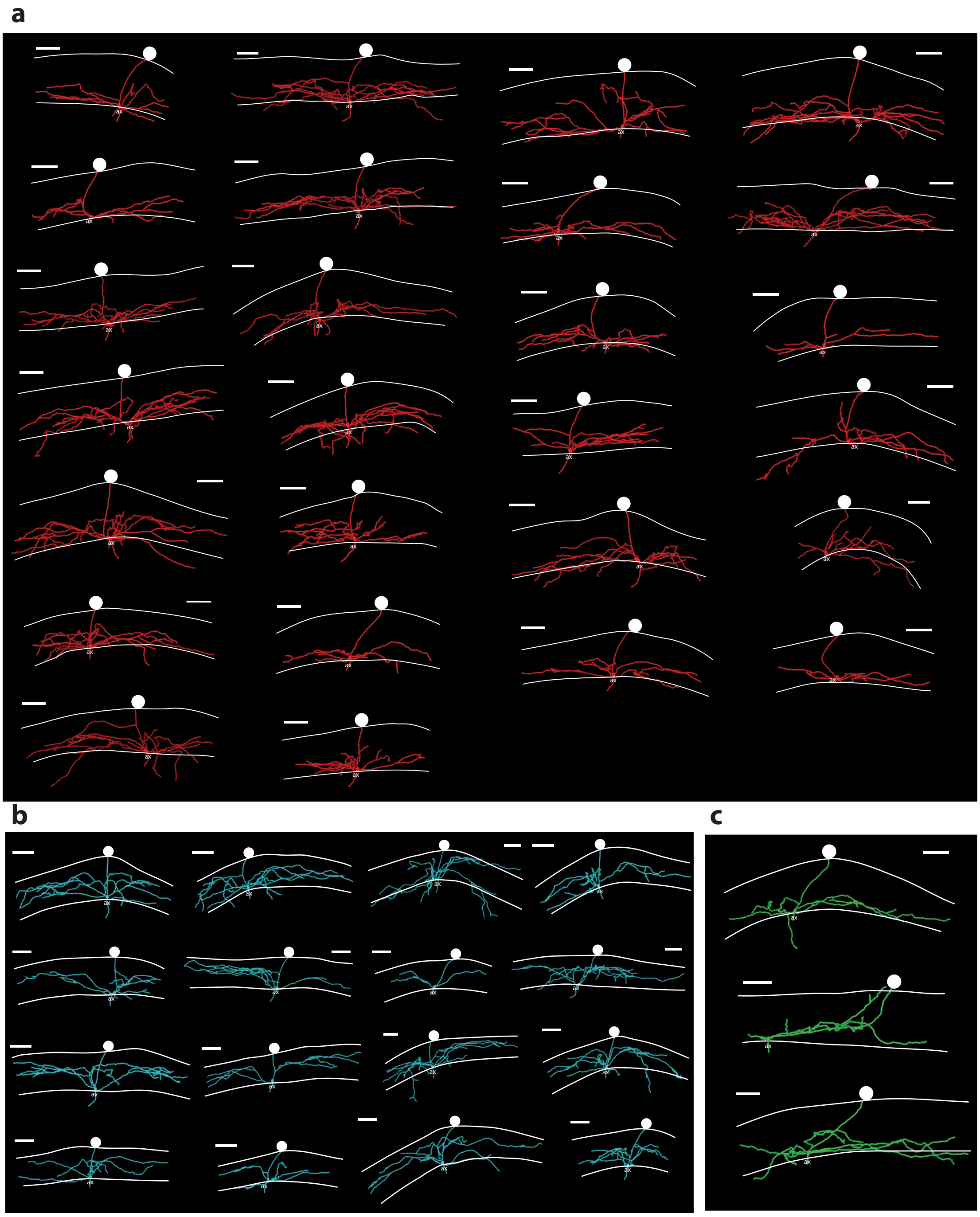

Supplementary Figure 4: Morphology galleries

Galleries showing dendritic morphologies of all identified and analyzed MCLPNs (**(a)** MCs, **(b)** dTCs, **(c)** sMCs). For each cell, the colored ovoid indicates the cell soma, the white circle the respective affiliated glomerulus. The upper white line adjacent to the glomerulus represents the GL-EPL border, the lower white line the MCL. 'Ax' denotes the initial segment of the axon. Scale bars = 100 µm.

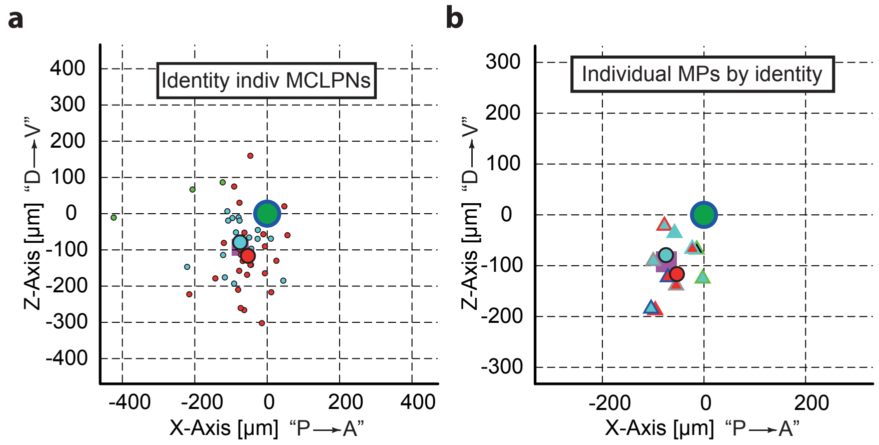

Supplementary Figure 5: Identity of MCLPNs

(a) Same representation of MCLPNs as in Fig. 5a after separation into MCs (small red dots), dTCs (small cyan dots) and sMCs (small green dots). **(b)** Individual MC (red triangles) and dTC (cyan triangles) midpoints (MPs) for each individual experiment (coloured lining reveals experiment identity). Large red and cyan points in a) and b) represent common MC and dTC midpoints, respectively. Magenta square indicate common MCLPN midpoint as determined in Fig. 5b. Large green circle illustrating the glomerular centre. Corresponding approximate anatomical orientations are also provided (A = anterior, P = posterior, V = ventral, D = dorsal, M = medial, L = lateral).

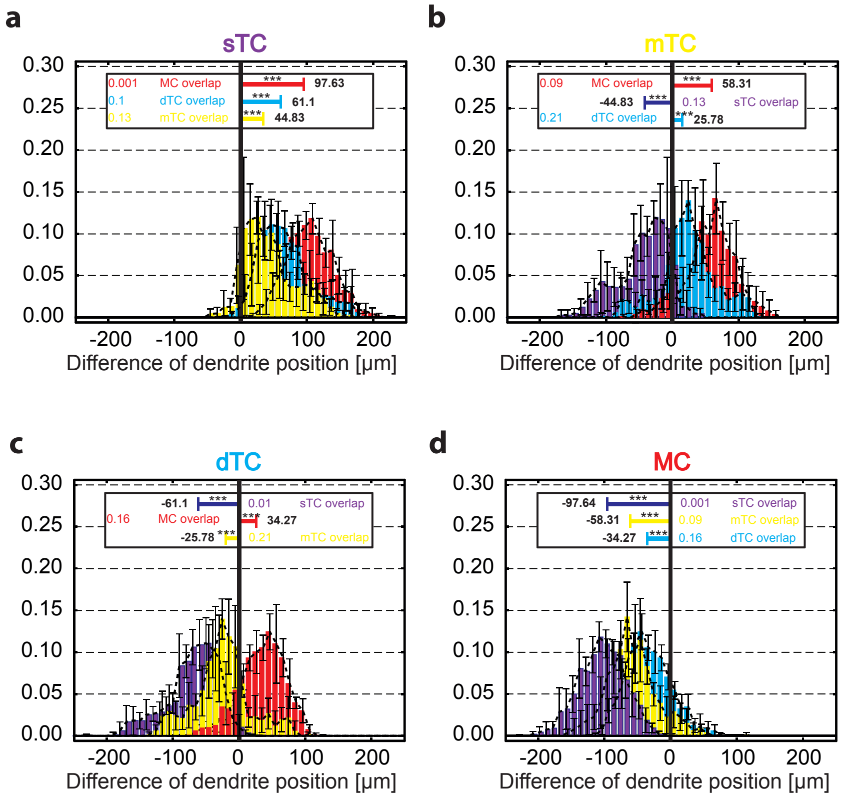

Supplementary Figure 6: Relative dendritic position differences between projection neurons

Dendritic reconstructions of the four cell types (**(a)** sTC, **(b)** mTC, **(c)** dTC and **(d)** MC) were considered separately, and bin-wise differences to the other three respective cell types calculated. Each cell type is marked by an individual colour: sTC = magenta, mTC = yellow, dTC = cyan and MC = red. For every cell type the distribution of mean positional differences is plotted as a histogram. X-axis represents the absolute distance between the considered cell type and the cell type indicated by the colour of the bars in µm. Negative values indicate a more superficial location and positive values indicate a deeper location of the bin. Y-axis represents the relative contribution of every bin. Standard deviations over five experiments for every bin are shown in black. Horizontal coloured bars illustrate the distance to the mean of the distribution, and the mean value is given. The cumulative 'overlap' (i.e. relative proportion of distances to the non-dominant side) is also indicated. All distance distributions are highly significantly shifted from 0 (one-sample t-test, in all cases $p < 0.001$ indicated by ***).

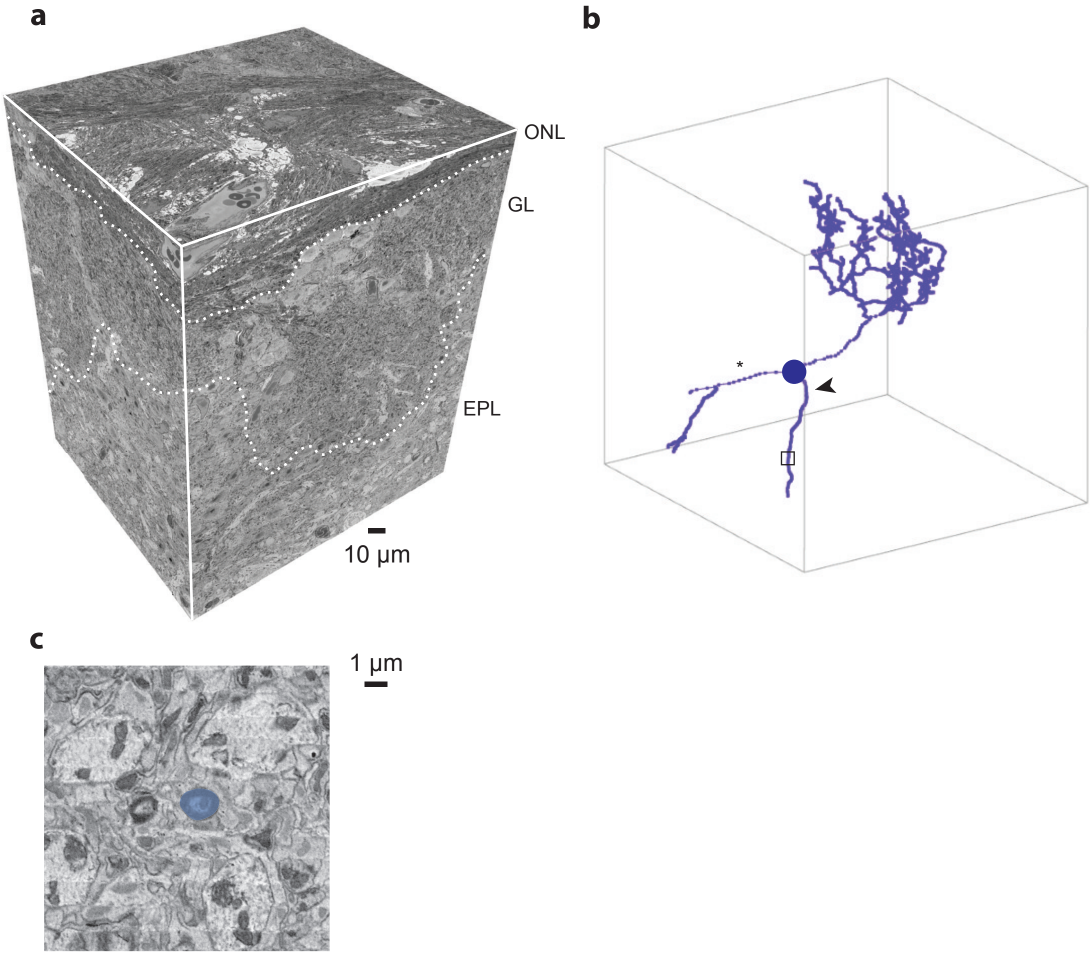

Supplementary Figure 7: EM identification of TC axons

TCs display myelinated axons that cross the EPL. SBEM dataset containing a glomerulus and adjacent EPL shown in **(a)**. TC neurons **(b)** were identified based on their characteristic cytoarchitectural features, namely a pale cytoplasm, a single apical dendrite that branches profusely in one glomerulus, and the presence of long-range lateral dendrites (asterisk). In some cases an axon was easily identifiable (arrowhead). This axon eventually became myelinated (box in b reported in **(c)**). Abbreviations: ONL, olfactory nerve layer; GL, glomerular layer; EPL, external plexiform layer.

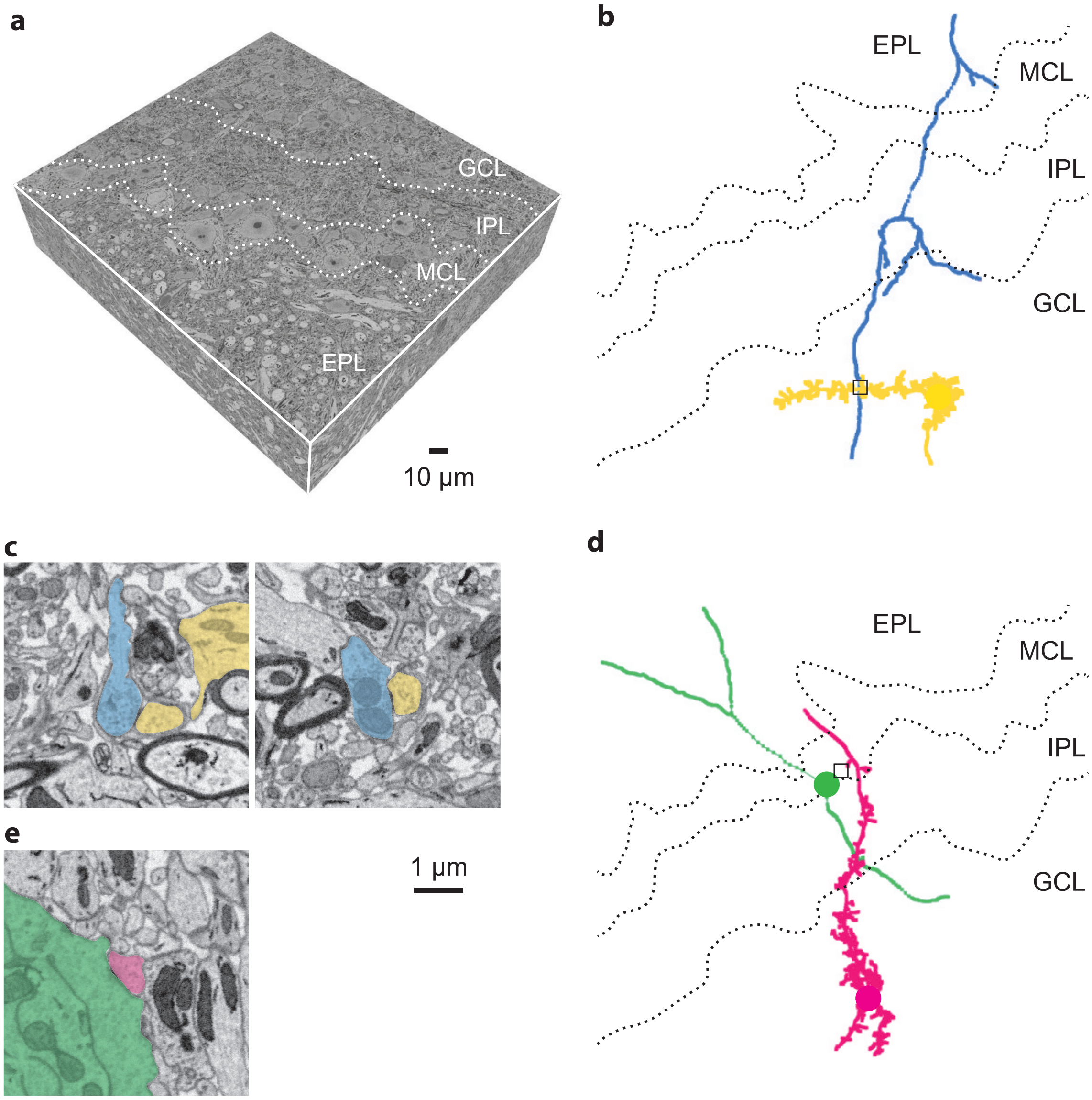

Supplementary Figure 8: EM identification of putative TC→GC axodendritic and GC→MC dendro-somatic synapses

EPL-originated myelinated axons establish synapses in the IPL onto GCs. SBEM dataset containing layers EPL, MCL, IPL and GCL **(a)**. Myelinated axons in the EPL were identified and traced as they entered the IPL **(b)**. These axons were found to establish axodendritic synapses (box in b reported in **(c)**) onto a granule cell (yellow). This dataset contained other granule cells (**(d)**, pink) that established synapses onto MC (box in d reported in **(e)**, MC in green). Abbreviations: EPL, external plexiform layer; MCL, mitral cell layer; IPL, internal plexiform layer; GCL, granule cell layer; MC, mitral cell; GC, granule cell.

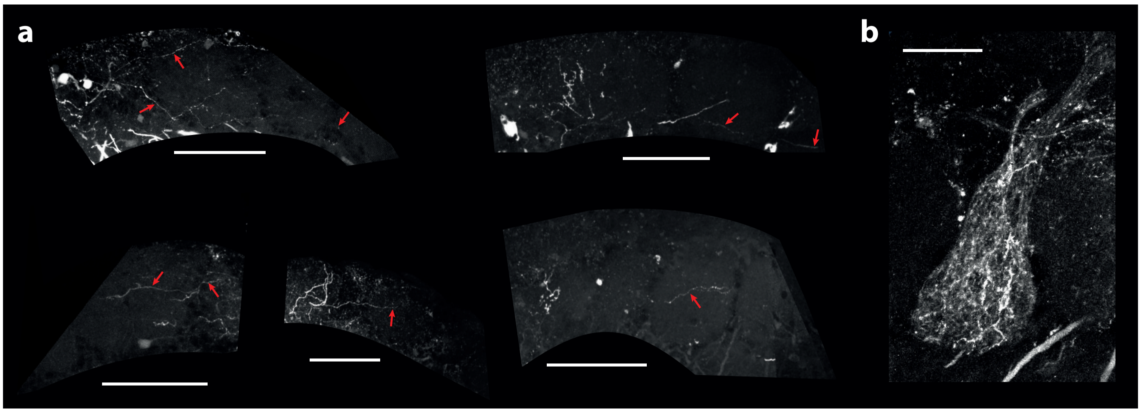

Supplementary Figure 9: Successful electroporation of thin processes of putative "juxtaglomerular association neurons" in distant glomeruli and olfactory sensory nerve axons.

(a) Red arrows indicate fine transglomerular axon-like processes resembling those of putative juxtaglomerular association neurons[38, 39] in glomeruli distant to the original electroporation site. Scale bars = 100 µm. **(b)** Olfactory nerve electroporation with subsequent labeling of the corresponding glomerulus. Scale bar = 50 µm.

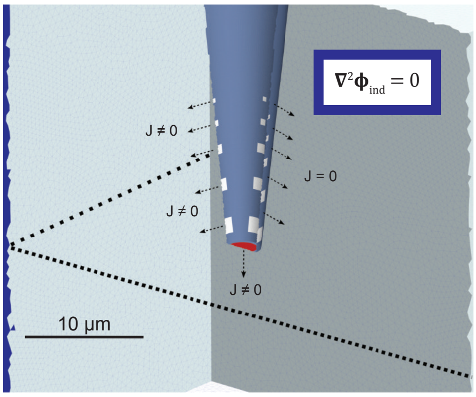

Supplementary Figure 10: Modelling landscape of the FEM simulation

Centre-cut of the modelling landscape, original pipette geometry, outline of calculation mesh elements and boundary conditions. J denotes the current density of the pipette surface which is 0 (arrows on the right) except for the holes and the tip (arrows on the left and bottom). Inset: Laplace's equation outside the electrode. All simulations include an assumed resting membrane potential of -70 mV.

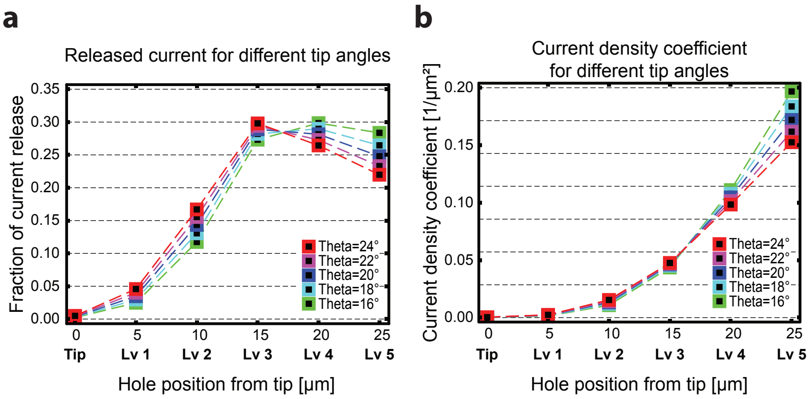

Supplementary Figure 11: Shape-dependent electrostatic behavior

(a) illustrates the dependency of the fraction of released current and **(b)** the current density coefficient at different hole levels from the tip angle theta (as indicated). The released current is more evenly distributed between the holes at shallower angles. Accordingly, the current density coefficient of distant holes to the tip is much smaller for shallower angles.

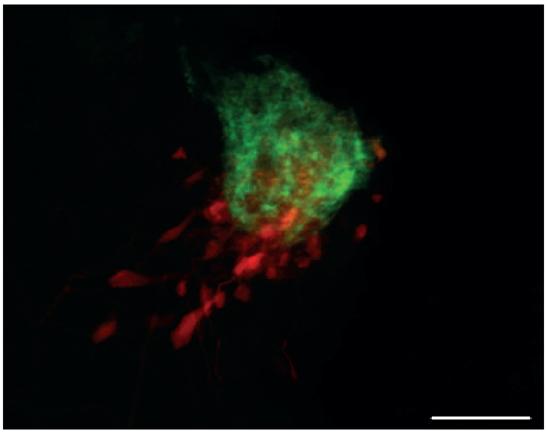

Supplementary Figure 12: Two-photon visualization of electroporated neurons *in vivo*

Two-photon projection of the *MOR174-9-GFP* glomerulus labeled by local electroporation of Alexa594 hydrazide. Scale bar = 50 μm.

a

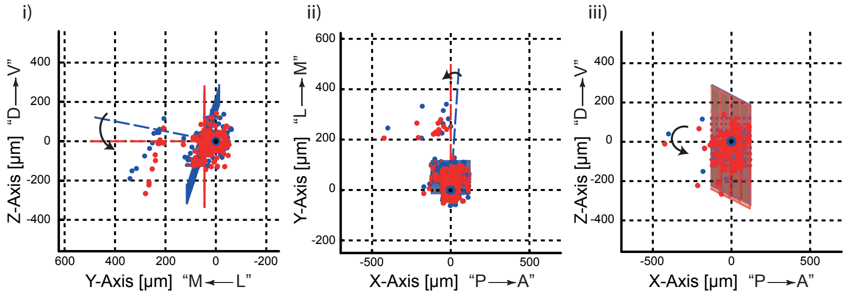

b

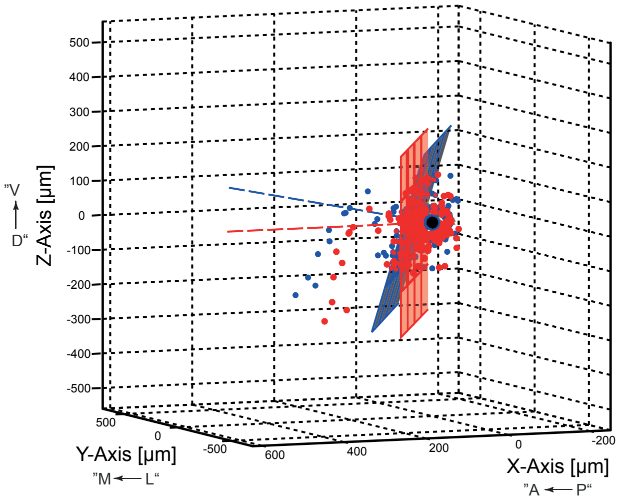

Supplementary Figure 13: Stepwise illustration of the morphing operation

(a) Illustration of the three basic consecutive rotation operations of a sample experiment (rotation around x-axis (i), rotation around z-axis (ii) and rotation around y-axis (iii)) transforming the 3D soma positions from one experiment to the common coordinate system. Black arrows indicate the direction of the rotation. **(b)** Overlay of soma positions before (blue) and after (red) the morphing operation of the same example experiment from a) in an oblique view of the 3D space. Dots represent soma positions, planes show the orientation of the fitted plane to the 9 x 9 point grid, and dashed lines indicate the central orthogonal axis through the plane and the midpoint of the glomerulus (black dot). Corresponding approximate anatomical orientations are also provided (A = anterior, P = posterior, V = ventral, D = dorsal, M = medial, L = lateral).

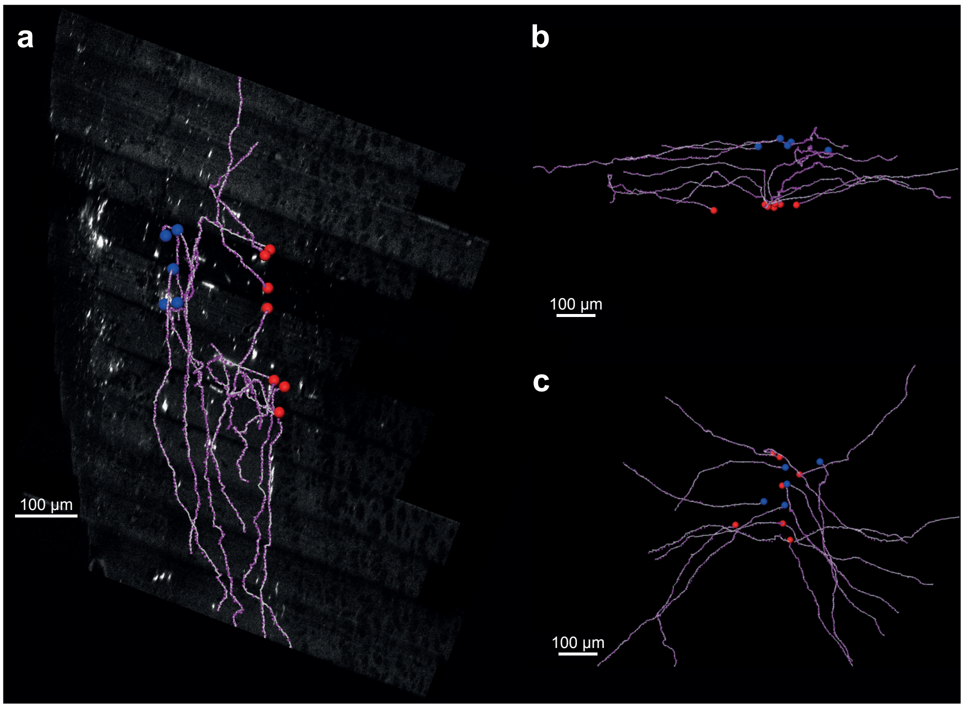

Supplementary Figure 14: Robustness of dendritic tracing

Example back-tracing experiment with the retrace shown by magenta dots. White lines represent dendritic trajectories of the original dataset, red dots show soma locations within the MCL while blue dots indicate soma locations in the superficial EPL. **(a)** shows a lateral (y-z) view with a detail overlay of the original data stacks outlining physical brakepoints. **(b)** and **(c)** indicate the corresponding orthogonal planes x-y and x-z, respectively.

Supplementary Discussion

The Total Number of Neurons per Glomerulus:

While neuron number likely varies significantly among glomeruli, many studies have tried to provide quantitative morphometric descriptions of various elements in the olfactory bulb across species[1-12]. However, most of these studies used global quantification methods in which the total population of glomeruli and/or the overall number of certain cell types were estimated. The composition of the glomerular domain was then calculated as an average ratio of cells per glomerulus. Moreover, cell type identification has only been based on bulbar layer identity of the cells and not on morphological parameters as in our study[7]. More specific quantitative approaches[12-14] have only become feasible in recent years with the arrival of targeted electroporation.

Since no systematic assessment of the quantitative extent of this technique has been undertaken to date, the exhaustiveness of the method is unclear but numbers between 7 and 16 MCL cells per glomerulus were reported in these studies. Other cell types have not yet been assessed quantitatively by this or a similarly direct technical approach.

Importantly, the total numbers of cells as well as the numbers of MCL cells per glomerulus of the earlier, global estimates and the specific targeted approaches differ substantially, i.e. at least by a factor of two. This difference might be attributable to an incomplete delineation of the population of glomerulus-associated neurons by the targeted electroporation approach, but relevant inter-observer variability in the assessment of global estimates may also play a role. However, an interesting recent study[6] provided convincing evidence that earlier global estimates in mice must be challenged due to an about twofold higher re-estimation of the total number of glomeruli per bulb compared to earlier studies[1, 2, 4] when using a new, potentially more rigorous approach based on immunohistochemistry that allows for reliable detection of small glomeruli. Such glomeruli had most likely been neglected in earlier studies resulting in a

systematic underestimation of the number of glomeruli[1, 2, 4]. Thus, existing global estimates of the number of cells per glomerulus are likely to be inaccurate and a ‘gold standard’ to achieve a reliable quantitative description of the neuronal elements of the glomerular circuitry does not exist. Additionally, some cell types of the bulb such as short axon cells and other local interneurons do not extend any cellular process into the glomeruli but would likely be contained in average neuron counts.

Taking these limitations into account, we feel confident that the average cell number per *MOR174-9* glomerulus of around 200 cells we find in our electroporation data (taking into account a ‘missed’ number of 20 %) might be a most direct estimate of cell number. This is lower than estimated previously[7] but their reported figure of 441 cells was based on an earlier total number of glomeruli[2] and when adjusted by the most recent count by Richard *et al.*[6], their global estimation lies in a similar range (~ 220 cells).

The impact of animal age may also have to be taken into account here: PGs are continuously replaced by adult born neurons in the SVZ[15]. While earlier studies have claimed neuronal stability with a balanced turnover rate of neurons in the GL[16], it was reported more recently that at least the subpopulation of dopaminergic PGs showed a relatively strong increase with age[17]. The authors of that study did not explicitly address the question whether this finding was due to a dynamic remodelling of subpopulations within an overall stable total population, or whether this was hinting at an overall growing population. Also, environmental sensory enrichment did enhance proliferation of neuronal precursors in the SVZ already[18] as well as survival of newborn neurons[19]. Therefore, the number of around 200 glomerulus-associated cells in the relatively young animals used here (< 2 months) might increase further with age and olfactory experience.

Taken together, while we acknowledge that a ‘ground truth’ neuronal number per glomerulus does not exist to date and our independent retest-approach provides only a simplifying

assumption of completeness, the numbers we provide are highly consistent with all indirect measures and theoretical considerations which we can find experimentally or in the literature.

Supplementary References Supplementary Discussion:

1. Royet, J.P., Distel, H., Hudson, R. & Gervais, R. A re-estimation of the number of glomeruli and mitral cells in the olfactory bulb of rabbit. *Brain Res* **788**, 35-42 (1998).
2. Royet, J.P., Souchier, C., Jourdan, F. & Ploye, H. Morphometric Study of the Glomerular Population in the Mouse Olfactory-Bulb - Numerical Density and Size Distribution Along the Rostrocaudal Axis. *J Comp Neurol* **270**, 559-568 (1988).
3. Royet, J.P., Jourdan, F., Ploye, H. & Souchier, C. Morphometric Modifications Associated with Early Sensory Experience in the Rat Olfactory-Bulb .2. Stereological Study of the Population of Olfactory Glomeruli. *J Comp Neurol* **289**, 594-609 (1989).
4. Pomeroy, S.L., LaMantia, A.S. & Purves, D. Postnatal construction of neural circuitry in the mouse olfactory bulb. *J Neurosci* **10**, 1952-1966 (1990).
5. Allison, A.C. & Warwick, R.T. Quantitative observations on the olfactory system of the rabbit. *Brain* **72**, 186-197 (1949).
6. Richard, M.B., Taylor, S.R. & Greer, C.A. Age-induced disruption of selective olfactory bulb synaptic circuits. *Proc Natl Acad Sci U S A* **107**, 15613-15618 (2010).
7. Parrish-Aungst, S., Shipley, M.T., Erdelyi, F., Szabo, G. & Puche, A.C. Quantitative analysis of neuronal diversity in the mouse olfactory bulb. *J Comp Neurol* **501**, 825-836 (2007).
8. Benson, T.E., Ryugo, D.K. & Hinds, J.W. Effects of sensory deprivation on the developing mouse olfactory system: a light and electron microscopic, morphometric analysis. *J Neurosci* **4**, 638-653 (1984).
9. Nawroth, J.C., Greer, C.A., Chen, W.R., Laughlin, S.B. & Shepherd, G.M. An energy budget for the olfactory glomerulus. *J Neurosci* **27**, 9790-9800 (2007).
10. Meisami, E. & Safari, L. A quantitative study of the effects of early unilateral olfactory deprivation on the number and distribution of mitral and tufted cells and of glomeruli in the rat olfactory bulb. *Brain Res* **221**, 81-107 (1981).
11. Panhuber, H., Laing, D.G., Willcox, M.E., Eagleson, G.K. & Pittman, E.A. The distribution of the size and number of mitral cells in the olfactory bulb of the rat. *J Anat* **140 (Pt 2)**, 297-308 (1985).
12. Liu, A., Savya, S. & Urban, N.N. Early Odorant Exposure Increases the Number of Mitral and Tufted Cells Associated with a Single Glomerulus. *J Neurosci* **36**, 11646-11653 (2016).
13. Sosulski, D.L., Bloom, M.L., Cutforth, T., Axel, R. & Datta, S.R. Distinct representations of olfactory information in different cortical centres. *Nature* **472**, 213-216 (2011).
14. Ke, M.T. & Imai, T. Optical clearing of fixed brain samples using SeeDB. *Current protocols in neuroscience / editorial board, Jacqueline N. Crawley ... [et al.]* **66**, Unit 2 22 (2014).
15. Carleton, A., Petreanu, L.T., Lansford, R., Alvarez-Buylla, A. & Lledo, P.M. Becoming a new neuron in the adult olfactory bulb. *Nat Neurosci* **6**, 507-518 (2003).
16. Mizrahi, A., Lu, J., Irving, R., Feng, G. & Katz, L.C. In vivo imaging of juxtaglomerular neuron turnover in the mouse olfactory bulb. *Proc Natl Acad Sci U S A* **103**, 1912-1917 (2006).
17. Adam, Y. & Mizrahi, A. Long-term imaging reveals dynamic changes in the neuronal composition of the glomerular layer. *J Neurosci* **31**, 7967-7973 (2011).
18. Alonso, M.*, et al.* Turning astrocytes from the rostral migratory stream into neurons: a role for the olfactory sensory organ. *J Neurosci* **28**, 11089-11102 (2008).
19. Alonso, M.*, et al.* Olfactory discrimination learning increases the survival of adult-born neurons in the olfactory bulb. *J Neurosci* **26**, 10508-10513 (2006).
